# The complete mitochondrial genome of the cryptic species in peanut worm *Sipunculus nudus* (Sipuncula, Sipunculidae) from Beibu Bay

**DOI:** 10.1080/23802359.2018.1463830

**Published:** 2018-04-23

**Authors:** Shengping Zhong, Yanfei Zhao, Qin Zhang, Xiuli Chen

**Affiliations:** a Key Laboratory of Marine Biotechnology, Guangxi Institute of Oceanology, Beihai, China;; b Guangxi Key Laboratory of Aquatic Genetic Breeding and Healthy Aquaculture, Guangxi Academy of Fishery Sciences, Nanning, China

**Keywords:** Mitochondrial genome, *Sipunculus nudus*, Sipuncula

## Abstract

The peanut worm *Sipunculus nudus* is an economic important species in the fishery and aquaculture in Indo-West Pacific. However, it is a species complex consisting of cryptic species, which can be difficult to identify based on morphology. Here, we report the complete mitochondrial genome sequence of *S. nudus* from Beibu Bay. The mitogenome has 15,375 base pairs and made up of total of 38 genes (13 protein-coding, 23 transfer RNAs, and two ribosomal RNAs), and a putative control region. There were 5948 mutations sites between Chinese and French populations. This study adds a distinct mitogenome of *S. nudus*, and will provide useful genetic information for future genetic variation identification and genetic diversity evaluation of this economic valuable marine benthic invertebrate.

Peanut worm (*S. nudus*) (Sipuncula, Sipunculidae) is a commercially important fisheries resource of high nutritional value with a global distribution (Hsu et al. 2013). Recent research has revealed the occurrence of cryptic species in this putatively cosmopolitan species (Hsu et al. 2013; Kawauchi and Giribet 2014). Due to limited set of external morphological and internal anatomical characters, misidentifications are often the case in *Sipunculus* (Kawauchi and Giribet 2014). In recent years, the wild stocks of *S. nudus*, however, have been overexploited to satisfy market demand because of high nutritional value (Lemer et al. 2015; Song et al. 2016). The artificial breeding technique of *S. nudus* has been developed in China since 2008, however, the stock enhancement programs need to be conducted for recovery of wild populations of *S. nudus*. Accurate species identification and genetic structure assessment of *S. nudus* are fundamental prerequisite for wild stock enhancement. Mitochondrial genome is a useful molecular technique for species delimitation, especially when there is small number of unambiguous taxonomic characters available for species delimiting. In this study, we report the complete mitochondrial genome sequence of *S. nudus* from Beibu Bay, which will be an important genetic resource to assist in species delimitation and resource management of *S. nudus*.

The tissue samples of *S. nudus* from five individuals were collected from Beibu Bay, China (Beihai, 21.439189 N, 109.297436 E), and the whole body specimens (#GG0029) were deposited at Marine biological Herbarium, Guangxi Institute of Oceanology, Beihai, China. The total genomic DNA was extracted from the muscle of the specimens using an SQ Tissue DNA Kit (OMEGA, Guangzhou, China) following the manufacturer’s protocol. DNA libraries (350 bp insert) were constructed with the TruSeq NanoTM kit (Illumina, San Diego, CA) and were sequenced (2 × 150 bp paired-end) using HiSeq platform at Novogene Company (Beijing, China). Mitogenome assembly was performed by MITObim (Hahn et al. 2013). The complete mitogenome of peanut worm from Zhangzhou, China (GenBank accession number: KJ754934) was chosen as the initial reference sequence for MITObim assembly. Gene annotation was performed by MITOS (Bernt et al. 2013).

The complete mitogenome of *S. nudus* from Beibu Bay was found to be 15,375 bp in length (GenBank accession number: MG873457), including the usual set of gene for mitogenome except for extra threonine tRNA which consists of 13 protein-coding, 23 tRNA, and two rRNA genes, and a putative control region. The overall base composition of the mitogenome was estimated to be A 29.3%, T 28.8%, C 27.2%, and G 14.7%, with a high A + T content of 57.5%, which is similar, but slightly different from Zhangzhou population (Song et al. 2016). The mitogenome of *S. nudus* shared 78.6% (3109 mutations) identities within Chinese populations, but 63.0% (5948 mutations) identities between Chinese and French populations. This result indicated the higher genetic divergence between Chinese and French populations, which further supports the occurrence of cryptic species in the peanut worm (Hsu et al. 2013; Kawauchi and Giribet 2014). The result of phylogenetic tree of Sipuncula also supported the closer relationship within Chinese populations than French populations, (Figure 1), as they shared the same branch node with the highest bootstrap value. The complete mitochondrial genome sequence of *S. nudus* from Beibu Bay, adds a distinct mitogenome of *S. nudus*, which will contribute to further comparative mitogenome studies and conservation of this economic valuable marine benthic invertebrate.

**Figure 1.**
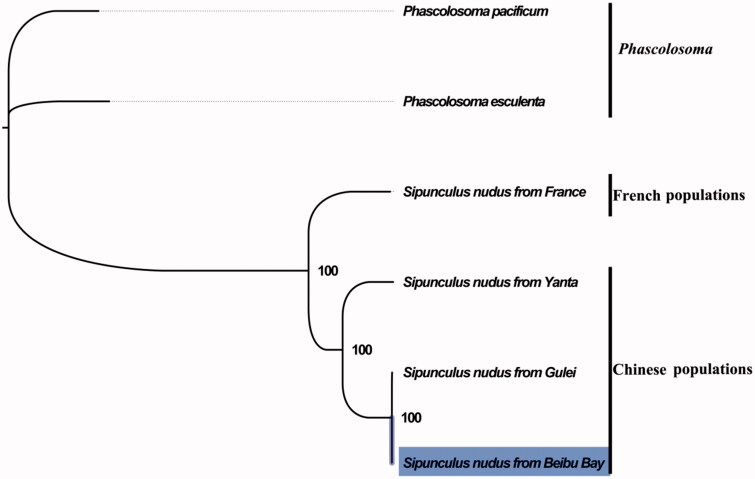
Phylogenetic tree in Sipuncula. The complete mitogenome is downloaded from GenBank and the phylogenic tree is constructed by maximum-likelihood method with 100 bootstrap replicates. The bootstrap values were labelled at each branch nodes. The gene’s accession number for tree construction is listed as follows: *Phascolosoma pacificum* (NC_031412), *Phascolosoma esculenta* (NC_012618), *Sipunculus nudus* from France (NC_011826), *Sipunculus nudus* from Yanta (KP751904), and *Sipunculus nudus* from Gulei (KJ754934).
